# NEIGHBORHOOD SOCIAL TIES AND LONELINESS IN RETIRED AND CURRENTLY WORKING OLDER ADULTS

**DOI:** 10.1093/geroni/igad104.0187

**Published:** 2023-12-21

**Authors:** Jaclyn Keenoy, Ashley Ermer

**Affiliations:** Montclair State University, Montclair, New Jersey, United States; Montclair State University, Montclair, New Jersey, United States

## Abstract

Peripheral social ties such as coworkers and neighbors have been identified as helpful to protect against loneliness, considering their convenient geographic proximity and ability to contribute diversity into one’s social network (Sandstrom & Dunn, 2014). However, the importance of neighborhood social ties for older adults has rarely been investigated by employment status. We anticipated that with an absence of coworkers to serve as peripheral social ties, retirees will rely more heavily on neighborhood social ties to alleviate feelings of loneliness. Using Wave 3 of the National Social Life, Health, and Aging Project, 1,088 currently working and 1,594 retired older adults were compared using path modeling in Mplus (v. 8). We first ran an overall model and found that there was a significant negative association between neighborhood social ties and loneliness among all participants (p=.000). Next, we ran a multigroup model between working and retired older adults. In this model, neighborhood social ties and loneliness were negatively associated for both retired (p=.000) and working (p=.047) respondents. A Wald test revealed that these groups were not significantly different (p=.100). Our results suggest that neighborhood social ties help to alleviate loneliness similarly in both working and retired older adults. This is despite those currently working likely having a network of coworkers as peripheral social ties from their workplaces. These findings contribute to the broader literature on social networks, and particularly advance the social convoy model’s understanding of the importance of peripheral social ties, even in older age (Huxhold et al., 2020).

